# Frontal Lobe Dysfunction and Impulse Control Problems as a Neuropsychiatric Manifestation of Progressive Multiple Sclerosis: A Case Report

**DOI:** 10.1192/j.eurpsy.2026.11030

**Published:** 2026-07-31

**Authors:** T. Üğüden, Y. Çiçek, N. G. Usta Sağlam, F. S. Bayır, M. Tütüncü, S. Turan

**Affiliations:** 1 Department of Psychiatry; 2 Department of Neurology, Istanbul University-Cerrahpasa , Cerrahpasa Medical Faculty, Istanbul, Türkiye

## Abstract

**Introduction:**

Multiple sclerosis (MS) is a chronic demyelinating disease of the central nervous system characterized by sensory and motor deficits and a wide spectrum of neuropsychiatric manifestations, including mood disorders, cognitive decline and behavioral disinhibition.Impulse control problems are rare but clinically relevant, as they may mimic primary neurodegenerative syndromes and complicate management.

**Objectives:**

We present a patient with MS with a progressive course and bilateral frontal lobe involvement manifesting impulsive and disinhibited behaviors. This case highlights the importance of recognizing MS-related neurodegeneration as a potential cause of dysexecutive syndromes and distinguishing it from frontotemporal dementia (FTD).

**Methods:**

Psychiatric assessment included a mental status examination, neuropsychological testing and the Barratt Impulsiveness Scale–Short Form (BIS-11-SF). Neurological evaluation comprised lumbar puncture and cranial MRI.

**Results:**

A 57-year-old man presented with three years of progressively worsening inappropriate spending, perseverative gambling, and compulsive hoarding. His psychiatric history included irritability and aggression treated in 2016, and he was most recently on escitalopram 20 mg/day and mirtazapine 30 mg/day.On examination, he displayed indifference, delayed responses due to word-finding difficulty, impaired attention, apathetic mood, restricted affect, and poor abstraction, while other cognitive functions were relatively preserved and no psychotic features were observed. Neuropsychological testing demonstrated slowed mental control, impaired attention and markedly reduced verbal fluency, consistent with frontal dysfunction. On the BIS-11-SF, he scored 36 out of 60. MS had been diagnosed in 2011 after persistent left leg weakness, confirmed by CSF analysis and MRI. Initial therapy with glatiramer acetate was switched to rituximab in 2016 due to radiological and clinical progression, achieving clinical stability.However, cranial MRI in 2022 revealed global cortical atrophy disproportionate to age, most prominent in the bilateral frontal lobes, with confluent subcortical, juxtacortical and periventricular demyelinating lesions (Image 1–3).These findings, attributed to the progressive course of MS, could explain his cognitive, emotional and behavioral symptoms associated with a dysexecutive syndrome.

**Image 1:**
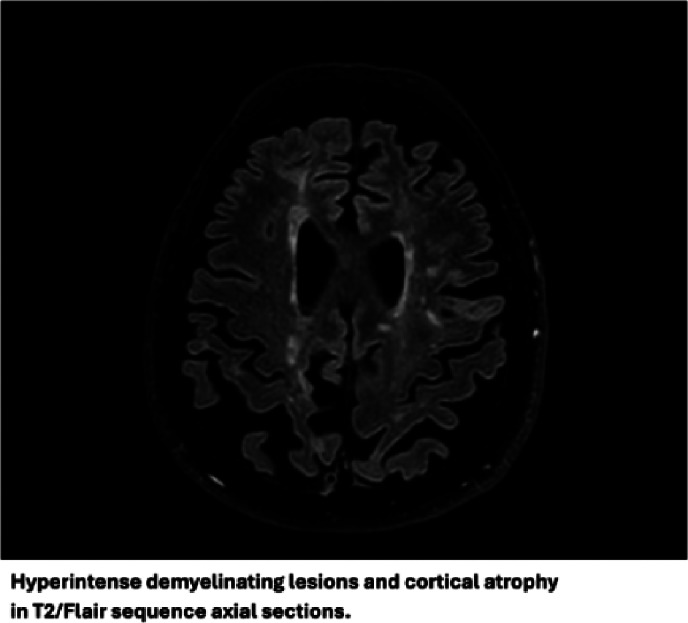
Image 1: Long description.

**Image 2:**
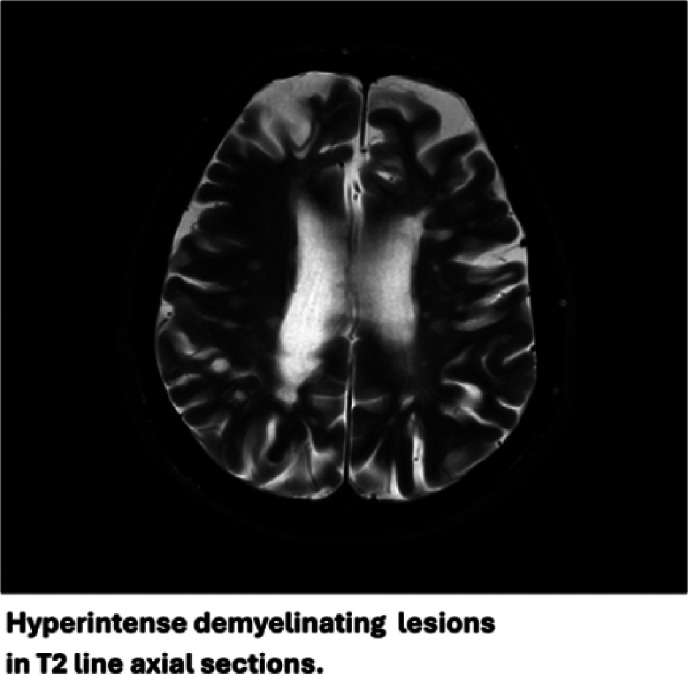

**Image 3:**
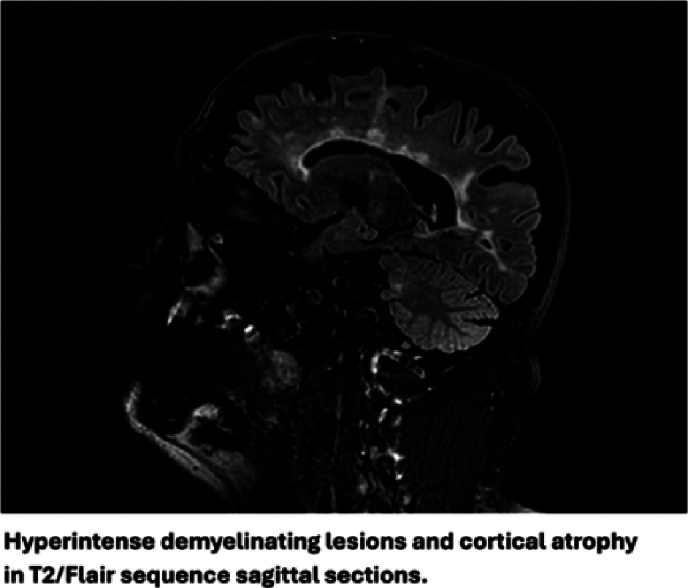
Image 3: Long description.

**Conclusions:**

MS with a progressive course may lead to cortical atrophy and neurodegeneration beyond demyelination, causing complex neuropsychiatric symptoms. In this case, frontal atrophy was associated with executive dysfunction, disinhibition and impulsivity. Radiological progression preceded behavioral symptoms and along with MRI evidence of widespread demyelination, supported MS-related neurodegeneration rather than FTD. Recognizing this distinction is essential for accurate diagnosis and appropriate management.

**Disclosure of Interest:**

None Declared

## Long descriptions

### Image 1: Long description

At the center of the axial brain section, the lateral ventricles appear as two dark, enlarged, butterfly-shaped voids, indicating ventriculomegaly. Surrounding the borders of these ventricles are bright, white hyperintense signals representing demyelinating lesions. Moving outward into the cerebral parenchyma, the white matter contains scattered smaller hyperintense spots. The cortical ribbon at the perimeter shows deep, widened sulci and thinned gyri, characteristic of advanced cortical atrophy. The outermost layer is the thin, dark outline of the cranium. The overall contrast is typical of a T 2 F L A I R sequence where fluid is suppressed (dark) and pathology appears bright.

Navigate back to Image 1:.

### Image 3: Long description

This sagittal cross-section of the head reveals several pathological features. At the center, the corpus callosum appears thinned. Surrounding the lateral ventricles and extending into the deep white matter are multiple bright, hyperintense spots indicating demyelinating lesions. The cerebral cortex shows signs of atrophy, characterized by widened sulci and narrowed gyri, creating a more pronounced space between the brain tissue and the skull. Moving inferiorly, the cerebellum and brainstem are visible, with the spinal cord extending into the neck. The F L A I R sequence suppresses fluid signals, making the bright lesions in the white matter stand out against the darker brain parenchyma.

Navigate back to Image 3:.

